# A Neglected Link Between the Psychoactive Effects of Dietary Ingredients and Consciousness-Altering Drugs

**DOI:** 10.3389/fpsyt.2019.00591

**Published:** 2019-08-16

**Authors:** Neil R. Smalheiser

**Affiliations:** Department of Psychiatry, University of Illinois College of Medicine, Chicago, IL, United States

**Keywords:** cacao, cocoa, TRPV1 receptor, anandamide, capsaicin, nutmeg, Ayahuasca, microdosing

## Introduction

“Microdosing” of lysergic acid diethylamide (LSD) and other psychedelic drugs refers to an increasingly popular practice in which individuals ingest 1/10th or less of the doses needed to produce overt hallucinations or subjective reports of perceptual distortions [e.g., 5–10 µg (1, 2)]. Generally, these small doses are taken repeatedly, every few days, and are reported to have long-term positive psychological effects including mood elevation (1), as well as subtle effects such as measurable changes in temporal perception (2). Microdosing of drugs which are not thought of as traditional psychedelics, e.g., cannabis, has also been described.

In the present article, I propose that it may be useful to think of ingestion of some common dietary ingredients as a form of microdosing. There are three parts to this hypothesis: First, that common dietary ingredients have psychoactive properties; second, that they converge (at least in part) to regulate molecular pathways shared by drugs that alter consciousness; and third, that *via* these mechanisms, combinations of dietary ingredients can produce synergistic potentiation of central nervous system (CNS) effects.

1. **Common dietary ingredients have psychoactive properties.** Even in their usual intake range, a variety of spices including vanilla, black pepper, cacao, chili peppers, cloves, saffron, cinnamon, ginger, nutmeg, and turmeric have been described as having mild effects on mood [reviewed in Ref. (3)].

2. **Regulation of endogenous cannabinoid system.** Although they do not all share a common primary mechanism of action, I note that, surprisingly, many plant-derived dietary ingredients have been reported to enhance the body’s endogenous cannabinoid system [reviewed in Refs. (4–6)]: For example, capsaicin, the active ingredient in hot peppers, is reputed to have euphoric properties. Capsaicin binds specifically to the vanilloid TRPV1 receptor and binds weakly to cannabinoid CB1 and CB2 receptors (7). In turn, activation of the TRPV1 receptor stimulates synthesis and release of the endogenous cannabinoid anandamide (8), which binds CB1 and CB2 receptors and is a weaker agonist at the TRPV1 receptor (9). As well, cacao and its derivatives cocoa and chocolate contain N-linoleoylethanolamide and N-oleoylethanolamide, compounds which inhibit anandamide breakdown (10), as well as variable amounts of anandamide (10, 11).

Other spices have been reported to enhance endogenous cannabinoids directly or indirectly as well: Although the compound responsible for nutmeg’s hallucinogenic effects at high doses is myristicin, certain nutmeg extracts inhibit anandamide breakdown (12). Curcumin, the active ingredient in the spice turmeric, has antidepressant effects in animal models, and chronic, but not acute, administration of 150 mg/kg curcumin significantly raised anandamide levels in a variety of brain regions (13). (E)-β-caryophyllene (BCP) is a major nonpsychotogenic component in the essential oil of *Cannabis sativa* and is also prevalent in oregano, cinnamon, and black pepper. BCP binds selectively to the cannabinoid CB2 receptor with nanomolar affinity and acts as an agonist (14). Another nonpsychotogenic component of *Cannabis*, cannabidiol (CBD), binds the TRPV1 receptor and inhibits anandamide turnover (15, 16). CBD has antidepressant-like effects in animal models (17–20). Note that the anandamide hydrolysis inhibitor URB597 also has antidepressant effects in mice and rats (21), suggesting that elevation of anandamide may at least partially explain mood elevating effects produced by these ingredients. Two additional components of black pepper are worth noting: Guineensine is an anandamide reuptake inhibitor (22), whereas piperine, the pungent component of black pepper, is yet another agonist at the TRPV1 receptor (23), which would presumably stimulate synthesis and release of anandamide (8).

A number of drugs, dietary caloric and fat intake, exercise, and other lifestyle interventions are also known to enhance the endogenous cannabinoid system in man [upregulating cannabinoid receptors, increasing anandamide or 2-arachidonoyl glycerol (2-AG) synthesis, or inhibiting their degradation] (24). Although this is viewed as overall indicative of a state of health, the endogenous cannabinoid system plays different roles as a function of diet and lifestyle; for example, in sedentary persons eating a high-carbohydrate, high-fat diet, it may foster obesity and metabolic syndrome (4).

3. **A “high” achieved from dietary ingredients? The case of cacao.** Recreational use of dietary ingredients may produce a frank state of intoxication. For example, snorting raw cacao powder is reported to produce a euphoric “high” (25). This has generally been attributed to its content of caffeine, theobromine, and phenylethylamine, but in view of the previous section, it is particularly interesting to ask whether chocolate/cocoa/cacao might also produce a cannabinoid “high.” di Tomaso et al.’s initial report that chocolate contains anandamide (10) was refuted by di Marzo et al. (11) because the content of anandamide was thought to be too low to be of consequence. However, to be fair, the scenario most likely to be associated with a cannabinoid “high” is not eating chocolate or even snorting cocoa powder but the ritual consumption of large amounts of cacao as performed by the Olmecs, Mayans, and Aztecs.

To my knowledge, no one has examined heirloom strains of cacao for their content of anandamide, which might potentially have been much greater than in currently cultivated strains. More importantly, it is well known that the Olmecs, Mayans, and Aztecs drank their cacao mixed with chili peppers and/or vanilla pods, but it has generally been assumed that these were added for flavoring or as folk medicine remedies (26). To my knowledge, no one has pointed out a possible connection between the indigenous cacao ritual drinks and the synergy of cacao with capsaicin (from peppers) and vanillin (from vanilla): Namely, capsaicin and vanillin (and anandamide itself) are all agonists of the TRPV1 receptor, which stimulates production and release of endogenous anandamide. When mixed with N-linoleoylethanolamide and N-oleoylethanolamide from cacao, which inhibit anandamide breakdown, the levels of endogenous anandamide are augmented further. When breakdown of anandamide is inhibited pharmacologically or genetically, anandamide is able to produce a state of intoxication similar to tetrahydrocannabinol in rodents and nonhuman primates (27–29). Thus, I suggest that chili peppers and vanilla were not chosen coincidentally or haphazardly as flavorings but precisely because, together, they were able to achieve high levels of anandamide. This hypothesis can be tested by examining whether levels of circulating anandamide increase following ingestion of raw cacao powder, especially in the presence of capsaicin or vanilla. The observation that some later recipes mixed cacao with black pepper (see previous section) is congruent with this hypothesis as well.

4. **Other possible examples of dietary combinations producing synergistic CNS effects.**

- Nutmeg is a popular spice which has effects on the nervous system [for example, it produces antidepressant-like effects in mice (30, 31)], and ingestion of larger amounts of nutmeg can produce a mescaline-like state of intoxication with hallucinations (32). Although I did not find any published study of combinations of dietary ingredients which can enhance the effects of nutmeg, interestingly, one self-experimenter claims that making a tea that includes both nutmeg, cinnamon, and cloves can have this synergistic effect—the cinnamon and cloves acting to prolong the effects of nutmeg by inhibiting breakdown of nutmeg’s active ingredients (including but not necessarily restricted to myristicin) (33).
- Curcumin, the essence of turmeric, is another popular spice with antidepressant-like effects in mice which acts as a monoamine oxidase (MAO) inhibitor, among other reported psychoactive, an anti-inflammatory, and antioxidant effects (34, 35). As stated in Ref. (35):
  Despite its reported benefits via inflammatory and antioxidant mechanisms, one of the major problems with ingesting curcumin by itself is its poor bioavailability, which appears to be primarily due to poor absorption, rapid metabolism, and rapid elimination. Several agents have been tested to improve curcumin’s bioavailability by addressing these various mechanisms. Most of them have been developed to block the metabolic pathway of curcumin in order to increase its bioavailability. For example, piperine, a known bioavailability enhancer, is the major active component of black pepper and is associated with an increase of 2000% in the bioavailability of curcumin. Therefore, the issue of poor bioavailability appears to be resolved by adding agents such as piperine that enhance bioavailability, thus creating a curcumin complex.
- Ayahuasca, another indigenous ritual drink, is prepared by mixing the bark or stems of a particular species of vine (Banisteriopsis caapi) with the leaves of a particular species of shrub (Psychotria viridis) in a specific manner—the shrub generates the unstable psychedelic compound N,N-dimethyltryptamine (DMT), whereas the vine contains MAO inhibitory compounds that prevent the breakdown of DMT, ensuring its oral bioavailability and its ability to produce a psychedelic state of consciousness (36). On the one hand, this is a very clear case in which one ingredient generates a psychedelic compound and the other prevents its breakdown. On the other hand, it is less clear whether these ingredients were originally ingested as part of the indigenous daily diet. The caapi vine is used by itself as a medicinal plant, chewed, smoked, or drunk as a tea, whereas the juice of P. viridis leaves is used medicinally for treatment of migraines.

5. **Dietary ingredients that prolong the effects of cannabis and psychedelic drugs.** Herbal supplements, medicinal plants, and foods have been reasonably well studied in terms of their effects on cognition and mood and their interactions with therapeutic drugs [e.g., Refs. (37, 38)]. Yet, to my knowledge, the peer reviewed literature contains few, if any, studies of how dietary ingredients may affect the peak, duration, or quality of the experience produced by cannabis and psychedelic drugs. In contrast, claims that cacao (or chocolate), spices, and other dietary ingredients can potentiate the effects of cannabis, mushrooms, and other psychoactive drugs are discussed widely on the internet, and several commercial combination products are available. Participants in the Ayahuasca ritual are given detailed dietary instructions beforehand in order to minimize unwanted interactions of this type (39).

## Conclusions

Although it is well accepted that foods can stimulate or calm the nervous system, it would initially sound strange to assert that common food ingredients can produce psychedelic effects. Yet, as discussed here, many of them modulate the endogenous cannabinoid system, and certain spices and medicinal plants contain hallucinogenic compounds such as DMT and myristicin. Here, I discussed four examples in which one ingredient contains psychoactive compounds, and is combined with other ingredients containing breakdown inhibitors, in order to potentiate synergistically the CNS effects. Perhaps, it is more appropriate to compare the effects of dietary ingredients and their combinations with ingesting subclinical doses of psychedelic drugs repeatedly every few days, or so-called microdosing (1, 2). Such a regimen may also allow chronic effects to persist and build, by sidestepping the activation of negative feedback loops which might be elicited by higher sustained doses (e.g., upregulation of anandamide leads to downregulation of CB1 and CB2 receptors).

The concept of dietary microdosing is potentially important because it points out two areas that deserve investigation but which have been neglected by the academic scientific community: First, I suggest that the potential psychological and psychiatric impact of dietary ingredients may be larger than previously appreciated, particularly when they are combined in specific ways. Many spices and medicinal plants have been studied individually in antidepressant assays and clinical studies but not, to my knowledge, as synergistic combinations. Apart from studying curcumin as an add-on therapy [e.g., Ref. (40)], I have not found any studies that have investigated whether dietary ingredients modulate the response of patients to antipsychotic agents. Second, it appears to be likely that dietary ingredients can modulate the affect the peak, duration, or quality of the experience produced by psychoactive drugs [including, but not limited to, cannabis and psychedelic drugs (41)]. I suggest that this topic is worth studying within the realm of psychiatric neuroscience rather than remaining in the purview of self-experimentation by enthusiasts (25, 33).

## Author Contributions

NS conceived, analyzed, and wrote the entire manuscript.

## Funding

NIH grant P01AG039347 supported the effort and open access publication fees.

## Conflict of Interest Statement

The author declares that the research was conducted in the absence of any commercial or financial relationships that could be construed as a potential conflict of interest.
